# A Machine Vision Anomaly Detection System to Industry 4.0 Based on Variational Fuzzy Autoencoder

**DOI:** 10.1155/2022/1945507

**Published:** 2022-03-16

**Authors:** Wei Jiang

**Affiliations:** Zhengzhou College of Finance and Economics, Zhengzhou 450000, China

## Abstract

From a technological point of view, Industry 4.0 evolves and operates in a smart environment in which the real and virtual worlds come together through smart cyber-physical systems. These devices that control each other autonomously activate innovative functions that enhance the production process. However, the industrial environment in which the most modern digital automation and information technologies are integrated is an ideal target for large-scale targeted cyberattacks. Implementing an integrated and effective security strategy in the Industrial 4.0 ecosystem presupposes a vertical inspection process at regular intervals to address any new threats and vulnerabilities throughout the production line. This view should be accompanied by the deep conviction of all stakeholders that all systems of modern industrial infrastructure are a potential target of cyberattacks and that the slightest rearrangement of mechatronic systems can lead to generalized losses. Accordingly, given that there is no panacea in designing a security strategy that fully ensures the infrastructure in question, advanced high-level solutions should be adopted, effectively implementing security perimeters without direct dependence on human resources. One of the most important methods of active cybersecurity in Industry 4.0 is the detection of anomalies, i.e., the identification of objects, observations, events, or behaviors that do not conform to the expected pattern of a process. The theme of this work is the identification of defects in the production line resulting from cyberattacks with advanced machine vision methods. An original variational fuzzy autoencoder (VFA) methodology is proposed. Using fuzzy entropy and Euclidean fuzzy similarity measurement maximizes the possibility of using nonlinear transformation through deterministic functions, thus creating an entirely realistic vision system. The final finding is that the proposed system can evaluate and categorize anomalies in a highly complex environment with significant accuracy.

## 1. Introduction

The systems that make up the industrial environment in the Industry 4.0 standard and those inherited from the existing infrastructure show enormous heterogeneity resulting in a massive number of different interfaces with different characteristics and security requirements [1]. Unfortunately, modern architectural standards do not determine the organization of heterogeneous systems based on the essential security requirements, which translates into a significant increase of the attack surface for possible cyberattacks [2]. It is important to emphasize that cyberattacks in the industrial environment can be implemented as a rearrangement of the operation of mechatronic equipment, the configuration of different signs or alerts, the omission of steps in the production process, and so on [1, 3].

The current situation focuses more on the human factor, the experience, and the opinion of experts, using assistive technology to assess and mitigate risks and threats. There should be in-depth human supervision and intervention by highly qualified staff for best results with this approach. On the other hand, the rapid development of computer systems has led to artificial intelligence mechanisms to solve complex problems without human intervention [4].

One of the critical areas of application of computational intelligence algorithms is the recognition of anomalies in real-time machine vision systems [5]. The detection of abnormalities is wholly related to recognizing patterns in a dataset that depicts different behaviors than expected [6]. The goal is to detect possible deviations while maintaining low false alarm rates. The activity is monitored in real time with a regular pattern when applying anomaly detection algorithms [7]. When a deviation is detected, the safety management mechanism is activated to investigate the incident further and take measures to deal with it.

In an industrial environment, anomaly detection is used to intelligently identify divergent behavior that could lead to mechanical failure or other adverse conditions. This process provides a robust security mechanism for industry hubs and business network systems within Industry 4.0 [2, 8].

An additional objective of anomaly detection is the immediate identification of irregular use, misuse, and abuse of industrial systems by external factors and equipment failure cases [9]. The industrial environment consists of scattered heterogeneous nodes that exchange information through a common automated communication infrastructure. In this context, the complexity increases exponentially as the number of interconnected systems expands [10].

The main problem in the environment mentioned above concerns the heterogeneity in detecting anomalies, which imposes the integration of intelligent machine vision systems in many industrial systems. The purpose of these applications is to alert cyber-physical systems when items outside of predetermined specifications appear on the production line so that corrective decisions can be made promptly to ensure product quality and productivity [3]. These systems are adaptive and can deal with the uncertainty of the environment in which they are applied. Similarly, with the integration of vision in the production process, it is possible to detect abnormalities through visual inspection in time, offering significant benefits, especially in construction problems or material failures [11].

This work aims to create a machine vision device to ensure the quality of metal components in the automotive sector, where Industry 4.0 standards are applied. In particular, the VFA algorithm is proposed, which can detect poor assembly alignment in gearboxes that may be due to cyberattacks [12]. The process of detecting these anomalies is achieved by using blurred entropy and Euclidean measurement of blurred similarity between samples, thus creating an entirely realistic and highly reliable machine vision system.

The ability of automated visual inspection on the production line to detect anomalies, given that it targets many industrial products, has been a constant research demand, thoroughly investigated by the research community, with significant developments depicted in the relevant literature [7, 13, 14].

The rest of the work includes Section 2, which provides an overview of the methods found in the literature and related to similar technical standardization.  Section 3 describes in detail the methodology of the proposed system. In contrast, Section 4 explains the scenarios for implementing the proposed approach. Finally, Section 5 summarizes the research conducted and presents the future objectives that can extend it.

## 2. Literature Review

The concept of anomaly detection using artificial intelligence has been approached with various methods from the research community because of the numerous challenges involved, such as the vast amount and diversity of data to be analyzed. In recent years, we observed that the community has been researching various autoencoder combinations to solve complex problems effectively [15, 16]. Because of the depth and richness of information, the universality of applications, and the difficulty of monitoring processes, this research is becoming increasingly important.

Zimmerer et al. [17] demonstrated an anomaly detection method for identifying and determining aberrant spots in medical imaging. They used a mix of density and reconfiguration-based anomaly detection algorithms, which did not require labeled data and allowed for sample-by-sample anomaly scoring and determination. They showed how to boost anomaly scores by using a context encoder and a variational autoencoder. In a variational autoencoder for pixel-wise anomaly localization, they added the posterior deviations (KL divergence) from the prior latent variable distributions. They also employed a variational autoencoder to combine the previous variations with the reconversion error to improve localization, achieving encouraging results with the potential to improve and speed up future medical picture review and assessment.

Lee et al. [2], in 2018, introduced a sparse representation framework for large-scale and high-dimensional data that builds dictionaries depending on the subspace of variational autoencoder (VAE). This autonomous framework injects minimal reconstruction into VAE, which is divided into two parts: secret data mapping and concise dictionary info generation. It can be used to uncover secret data and extract more high-level features than hand-crafted features in large-scale datasets by acting as a dimensionality reducer.

Carletti et al. [18] proposed a method for determining “feature importance” in anomaly detection, with the goal of addressing the barriers to ML adoption in Industry 4.0 scenarios. The absence of supervised datasets makes intelligent monitoring systems difficult. The feature point importance evaluation method is intended for isolation forest, among the most widely used anomaly detection methods.

Banifakhr and Sadeghi [19] demonstrated a method for detecting anomalies in trajectories using CCTV records of vehicle traffic. The method makes use of machine learning and deep learning techniques to overcome the problems of not having enough data to build an effective model and not having enough anomaly data to cover all conceivable aberrant trajectories. They solved the challenge by combining optimal convolutional neural network and adaptive neuro-fuzzy inference system network classifiers with an autoencoding network to create an optimized structure for anomaly detection at the decision level. The classifier first categorizes the input trajectory into one of the specified groups. The result is then evaluated by the trained autoencoder networks to determine whether the route is regular or aberrant.

Tsai and Jen [14] sought to detect surface defect irregularities using an autoencoder. On the one hand, they did not use pixel-wise flaw separation, but instead used photo detectability. A normalization was included in the suggested convolutional autoencoder, which enhances the characteristic dispersion of fault examples within a small spectrum. This method brings all training samples' representative feature vectors as nearly as possible to the average feature vector. In the evaluation stage, a defect sample can produce a different range from the learned center of fault samples. They also added two normalization penalties that could limit the spread of retrieved properties from a group of fault samples to a small area. For less-regular texture backdrops, the first regularization is learned for uniformly surface areas, while the second could further differentiate the faulty features.

For evaluating ultrasonic testing (UT) data, Milkovic et al. [15] suggested a variational autoencoder (VAE). In standard UT data, the VAE was applied to characterize the distributions. Their strategy was to train on normal data only, which resulted in variations in VAE output and latent values in cases with abnormal data, which served as a foundation for anomaly identification. The problem of detecting anomalies in ultrasound pictures necessitated the use of numerous criteria. First, they rebuilt the error and variances of the mean and standard deviation of latent variable parameters. Then, on top of the decoder, they added a second encoder, allowing the use of two new parameters, which merged reconstructions and hidden descriptions as potential anomaly signs.

Finally, in 2022, Lu et al. [7] introduced a deep learning-based anomaly detection method for identifying lace faults in industrial settings. Lace is unique in that it is one of the only industrial items that is completely dependent on manual fault control. Video preprocessing, pixel rebuilding, and pixel categorization were the three stages of their system. Only defect-free lace films are required during the offline phase to train the pixel reconstruction model and determine the detection threshold using the adaptive thresholding method. The proposed framework reconstructs lace videos and conducts defect inspection utilizing reconstruction error and a predetermined threshold in the online stage. On holes and damaged yarn, their model worked perfectly. To overcome the dataset deficit, they aimed to gather more faulty samples, which is a time-consuming technique, and analyze the lace pattern layout. They also aimed to explore the pixel reconstruction model to obtain more precise rebuilding findings, which can help distinguish small problems and noises.

From the above literature, we can conclude that the research community is primarily trying to find a practical machine learning approach to solve complex problems with the most effective methods.

## 3. Proposed Machine Vision-Based Anomaly Detection System

In the present work, a holistic approach to anomaly recognition in machine vision systems is implemented and proposed, based on an original VFA methodology where the possibility of using nonlinear transformation through deterministic functions is maximized. This is an innovative model of artificial vision, for optimal decision making, regarding the recognition of anomalies in the industrial environment. Specifically, we present a novel methodology using fuzzy entropy and Euclidean fuzzy similarity measurement for the first time in the literature, in order to maximize the possibility of using nonlinear transformation through deterministic functions, thus creating an entirely realistic and highly reliable machine vision detection system for Industry 4.0 based on variational fuzzy autoencoder [15, 20].

**Figure 1:**
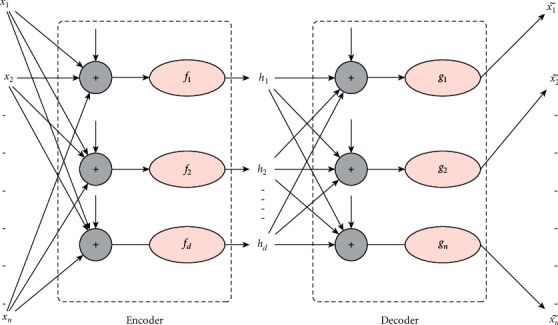
Autoencoder.

The evaluation of the methods was carried out in a highly complex cybersecurity scenario, where cybercriminals could modify the assembly parameters of the production mechanisms. This fact is not perceived by the other sensors connected to the production system. Utilizing the most advanced machine vision techniques and fuzzy logic methodologies, the proposed method has achieved very high success rates, creating serious expectations for additional cybersecurity applications [20, 21]. A depiction of the autoencoder architecture is presented in Figure 1.

The VFA architecture layout contains a hidden layer consisting of *D* neurons. The encoder encodes the input vector *x* into the vector *h*. Each *h*_*i*_ coordinate corresponds to the output of a hidden layer neuron so that

(1) $$\begin{array}{c} h_{i}=f_{i}\left(w_{ei}^{T}x+b_{ei}\right), \end{array}$$

where *f*_*i*_ is the activation function and *w*_*ei*_ and *b*_*ei*_ are the parameters of the *i*th neuron of the encoder. The decoder then decodes the representation by producing

(2) $$\begin{array}{c} {\tilde{x}}_{i}=g_{i}\left(w_{di}^{T}h+b_{di}\right), \end{array}$$

at output *i*, where *g*_*i*_ is the activation function and *w*_*di*_ and *b*_*di*_ are the parameters of the *i*th neuron of the decoder. The training is done by minimizing the loss function:

(3) $$\begin{array}{c} J\left(x,g\left(f\left(x\right)\right)\right). \end{array}$$

An easy way for the encoder to learn valuable features is through the *D* *<* *N* constraint, i.e., the dimensionality of the hidden representation is less than the dimensionality of the data. In this case, the encoder encrypts any incoming information, and then the decoder tries to reconstruct the input. Because *D* *<* *N* is valid, some of the information contained in the attribute space is lost. The decoder attempts to recover the lost data through *h*. The network, therefore, tries to trap as much information as possible in vector *h*, neglecting potentially useless information contained within the attribute space. If each *x*_*i*_ comes from an independent and identically distributed (iid) distribution independent of the others, then *h* rarely contains any helpful information. However, if there is any structure between the data, the autoencoder can detect it.

Another way to export useful features is by applying sparse restrictions to the network. For this purpose, additional constraints are introduced in the loss function that forces the network's neurons to be activated less frequently so that the *h*_*i*_ are as detachable as possible. Typical limitations concern the matrix of network weights, such as the norm *L*_1_ or *L*_2_. The parameter *λ* corresponds to a hyperparameter of the network, which is determined during its training. High values of the parameter give further power to the constraint by reducing the values of the network weights:

(4) $$\begin{array}{c} J\left(x,g\left(f\left(x\right)\right)\right)+\lambda \Omega \left(h\right). \end{array}$$

The proposed VFA methodology assumes some unknown distribution on the data to determine the distribution parameters. More specifically, let the dataset *X*={*x*^(*i*)^}_*i*=1_^*N*^, consisting of *N* iid samples. Each sample *x*^*(i)*^ comes from a random process of an unobservable random variable *h* which comes from some prior distribution *p*_*θ*_^*∗*^(*h*) so that from this distribution, a sample *h*^*(i)*^ is obtained, respectively, and a sample *x*^*(i)*^ is obtained from the bounded distribution *p*_*θ*_^*∗*^(*x|h*).

The process of giving birth to the samples comes from its separate latent variable, which it does not share with any other sample, i.e., there are no global latent variables. Based on the above hypothesis, the goal of the proposed system is to determine *p*_*θ*_^*∗*^(*x|h*). Because the random variables and the distribution parameters are unknown, according to Bayes theorem, the requested probability is the following:

(5) $$\begin{array}{c} p_{\theta^{*}}\left(h|x\right)=\frac{p_{\theta^{*}}\left(x|h\right)p\left(h\right)}{p_{\theta^{*}}\left(x\right)},\\ p_{\theta^{*}}\left(x\right)=\int p_{\theta^{*}}\left(x|h\right)p_{\theta^{*}}\left(h\right)\text{d}h. \end{array}$$

According to the above, the requested posterior is approached through a family of distributions. It is calculated based on the Kullback–Leibler divergence metric estimate, which quantifies the similarity between different distributions. Using the product of the logarithm to the common probability *p*(*h*, *x*) of the above equation, a parametric solution can take the following form:

(6) $$\begin{array}{c} J\left(\lambda \right)=\sum_{i=1}^{N}J_{i}\left(\lambda \right)\\ =\sum_{i=1}^{N}E_{q_{\lambda }\left(h|x_{i}\right)}\left\{\log\left(p\left(h_{i}|x_{i}\right)\right)\right\}-KL\left\{q_{\lambda }\left(h|x_{i}\right)\left\|\right)p\left(h\right)\right\}. \end{array}$$

Expressing the maximization problem as a minimization problem, the loss function of the proposed system can be described:

(7) $$\begin{array}{c} J\left(X,\theta ,\phi \right)=\sum_{i=1}^{N}J_{i}\left(x_{i},\theta ,\phi \right),\\ J_{i}\left(x_{i},\theta ,\phi \right)=-E_{h_{i}∼q_{\theta }\left(h_{i}|x_{i}\right)}\left\{\log\left(p_{\phi }\left(x_{i}|h_{i}\right)\right)\right\}+KL\left\{q_{\theta }\left(h_{i}|x_{i}\right)\left\|\right)p\left(h_{i}\right)\right\}. \end{array}$$

An issue that arises is the sampling process so that this selected version of the sample is as close as possible to the original. On the other hand, it is also required that the sampling requires computational time to be used in real applications. Our proposal for smoothing purposes is for selection to take place at a particular time for each new scale parameter and the required sample number. With this sampling policy, we avoid the repetition of a computationally demanding operation several times in each step since the samples are reused for different values of *x*. On the other hand, we guarantee the consistency of the property of triangular inequality that satisfies every norm, which can be applied as follows:

(8) $$\begin{array}{c} \hat{g}\left(x\right)=\frac{1}{n}\sum_{i=1}^{n}J\left(x-v_{i}\right),\;v_{i}∼Ν\left(0,\sigma_{k}^{2}I\right),\\ \hat{g}\left(y\right)=\frac{1}{n}\sum_{i=1}^{n}J\left(x-v_{i}\right),\;v_{i}∼Ν\left(0,\sigma_{k}^{2}I\right). \end{array}$$

The difference between the two equations is

(9) $$\begin{array}{c} \hat{g}\left(y\right)-\hat{g}\left(x\right)=\frac{1}{n}\left(\sum_{i=1}^{n}J\left(y-v_{i}\right)-\sum_{i=1}^{n}J\left(x-v_{j}\right)\right)\\ =\frac{1}{n}\left(\sum_{i=1}^{n}J\left(y-v_{i}\right)-J\left(x-v_{i}\right)\right),\;\left(i=j\Rightarrow v_{i}=v_{j}\right), \end{array}$$

so it applies to the norm:

(10) $$\begin{array}{c} \left\|\hat{g}\left(y\right)-\hat{g}\left(x\right)\right\|=\frac{1}{n}\sum_{i=1}^{n}J\left(y-v_{i}\right)-J\left(x-v_{i}\right)\\ \leq \frac{1}{n}\sum_{i=1}^{n}J\left(y-v_{i}\right)-J\left(x-v_{i}\right)\\ \leq \frac{1}{n}\sum_{i=1}^{n}L\left\|y-x\right\|\\ \frac{\left\|\hat{g}\left(y\right)-\hat{g}\left(x\right)\right\|}{\left\|y-x\right\|}\leq L. \end{array}$$

It is evident that if we selected new samples for each point, the algorithm might have failed to find the total minimum, primarily if it used first-order methods like slope descent, as the function is not smooth. This case substantially limits the acceptable cost functions that we can consider for optimization, as it requires the values of the function to be constrained. So, essentially, for a given required approach accuracy, the probability of adhering to it improves exponentially by increasing the number of samples.

Accordingly, given that uncertainty is directly related to the number of data samples, the amount of data about a state expresses the complete possible information. So, reducing the uncertainty since we have similarities between different distributions indicates an equal gain in the amount of data. The degree of similarity, in this case, expresses the degree of proximity of an element of *p*_*θ*_^*∗*^(*h*) compared to the original elements of the bounded distribution *p*_*θ*_^*∗*^(*x|h*). This interpretation is used to extract an abstract representation from a dataset, taking advantage of the proximity between different amounts of data. Furthermore, the above interpretation is used in the vague control. The degrees of similarity between the current and the reference situations in the rules form the basis for the interpolation mechanism between the conclusions.

Fuzzy entropy was used as a measure of the ambiguity of the whole, which results from the inherent ambiguity and vagueness of the whole itself due to the inability to separate the whole from its complement, that is, the abnormal elements from the normal. In this sense, the measures that assess uncertainty in the context of fuzzy set theory, namely, the entropy measures and the ambiguity indices, were adopted to implement a fully functional and realistic system for detecting anomalies in machine vision systems. The fuzzy entropy equation used is shown below:

(11) $$\begin{array}{c} E_{LT}^{FS}\left(\bar{A}\right)=-\frac{1}{n}\sum_{i=1}^{n}\left[\mu_{A}\left(x\right)\log_{2}\mu_{\bar{A}}\left(x\right)+\left(1-\mu_{\bar{A}}\left(x\right)\right)\log_{2}\left(1-\mu_{A}\left(x\right)\right)\right]. \end{array}$$

To measure the distance between normal and abnormal cases, the ambiguity index was used using the Euclidean distance:

(12) $$\begin{array}{c} f\left(\bar{Å}\right)={\left(\sum_{i=1}^{n}{\left(\mu_{\bar{A}}\left(x\right)-\mu_{\tilde{A}c}\left(x\right)\right)}^{2}\right)}^{1/2}. \end{array}$$

**Figure 2:**
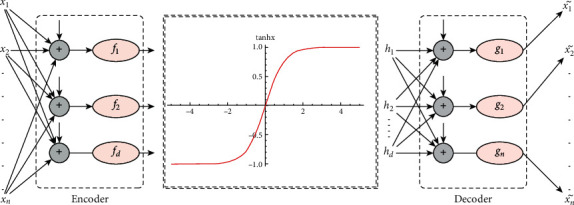
Variational fuzzy autoencoder.

Thus, the final shape of the proposed architecture takes an intermediate level at which fuzzy logic is applied to the investigation of anomalies. The proposed architecture is schematically presented in Figure 2.

## 4. Scenarios

The reliability of steel industry applications is an important growth factor in the shipping industry, aviation, defense systems development, renewable energy sources, etc. A typical example is the durability and accuracy of the operation of gearboxes, where the reliability of the assembly ensures their smooth and long-lasting operation. For a gearbox to work correctly, it must be precisely aligned with the power units, axles (e.g., driveshaft), and other components (e.g., differential) involved. When the other component and the gearbox are not connected properly, the gearbox is not aligned. Poor alignment puts a lot of pressure on the gearbox parts, such as the axles and the coupling, and can deplete the device enough to cause severe wear and even drive failure. When misaligned, one end of a gear can crack or open earlier than it should, and similar damage can occur to bearings.

Poor alignment can occur due to static factors such as manufacturing defects or user error. Dynamic causes include heavy loads stretching the gearbox components and thermal expansion. Also, other factors can cause poor alignments, such as tilt error or oscillation and centrifugal forces. A particular cause that can create misalignment is the improper configuration of SCADA control systems that control the production process through cyberattack.

During regular operation, the control unit operator monitors the standard operation variables of the assembly system on the production line provided by the corresponding sensor. Abnormal behavior occurs when some assembly parameters are not within the normal range. During assembly, a laser shaft alignment system can measure the misalignment of the gears and rotate them to take the correct position. This process is activated after a specific notification of the control system. A cyberattack could deactivate if the cybercriminals could modify the assembly parameters outside of the normal operating range. The proposed mechanical vision system augmented by the VFA algorithm is used to deal with this type of cyberattack, not connected to the production line system operating autonomously as an independent security mechanism.

For the implementation of the experiments, a dataset including snapshots with the operating condition of a component was used, where the primary anomaly is related to a specific type of error related to the alignment of the gearbox gears.

To address the problem of image matching, a heuristic algorithm was used to calculate the digital variation and then apply methods to repeat and optimize these values while calculating the fuzzy sample entropy and Euclidean fuzzy similarity. This method essentially captures pixel layout and smoothness, which minimizes pixel mismatches. Specifically, the calculation of the digital variation is done by minimizing an energy function *E*(*d*) as follows:

(13) $$\begin{array}{c} D=\arg\min\left(E\;{\;}_{d}\left(d_{p}\right)\right). \end{array}$$

The energy function consists of two terms. The first refers to the data and measures how well the variation function *d* matches the pair of images. The second term refers to the assumptions made by the algorithm:

(14) $$\begin{array}{c} E\left(d\right)=E_{\text{data}}\left(d\right)+\lambda E_{\text{smooth}}\left(d\right). \end{array}$$

The term *E*_data_ is equal to the sum of each pixel of the matching costs *C* of the disparity space image table:

(15) $$\begin{array}{c} E_{\text{data}}\left(d\right)=\sum_{\left(x,y\right)}C\left(x,y,d\left(x,y\right)\right). \end{array}$$

The term Ε_smooth_ is equal to the sum of the depreciation of the variation differences between adjacent pixels:

(16) $$\begin{array}{c} E_{\text{smooth}}\left(d\right)=\sum_{\left(x,y\right)}p\left(d\left(x,y\right)-d\left(x+1,y\right)\right)+p\left(d\left(x,y\right)-d\left(x,y+1\right)\right), \end{array}$$

where *x* is the scan column and *y* is the scan bar. The variable *p* is a function of the difference of the digital variant, genuinely increasing. The term *E*_smooth_(*d*) can be transformed to accommodate volume differences. This has the effect of reducing the smoothness of the image when the intensity gradient is high. The term *λ* is the relative weight of the normality term, and its value depends on the calculation method of the correlation cost.

Also, for the calculation of the correlation cost between two pixels using linear interpolations in the neighboring pixels, we used a parametric method which is more efficient and less sensitive to the effect of image signal sampling in case the brightness of the pixels changes abruptly, for example, in-depth discontinuities and repetitive patterns. The calculation was based on the following function:

(17) $$\begin{array}{c} C_{BT}\left(p,d\right)=\min\left(A,B\right),\\ A=\max\left(\begin{array}{c} 0,I_{L}\left(p\right)-I_{R}^{\max}\left(p-d\right),\\ I_{R}^{\min}\left(p-d\right)-I_{L}\left(p\right) \end{array}\right),\\ B=\max\left(\begin{array}{c} 0,I_{R}\left(p-d\right)-I_{L}^{\max}\left(p\right),\\ I_{L}^{\min}\left(p\right)-I_{R}\left(p-d\right) \end{array}\right),\\ I^{\min}\left(p\right)=\min\left(I^{-}\left(p\right),I\left(p\right),I^{+}\left(p\right)\right),\\ I^{\max}\left(p\right)=\max\left(I^{-}\left(p\right),I\left(p\right),I^{+}\left(p\right)\right),\\ I^{-}\left(p\right)=\frac{\left(I\left(p-{\left[10\right]}^{T}\right)+I\left(p\right)\right)}{2},\\ I^{+}\left(p\right)=\frac{\left(I\left(p+{\left[10\right]}^{T}\right)+I\left(p\right)\right)}{2}. \end{array}$$

Finally, we used the normalized correlation coefficient, which is the normalized expression of the variability of the reference windows and the search of the contrasted images. This coefficient is calculated using the average and the standard deviation of the values of the intensity of the brightness in the window. In this work, the correlation coefficient remains unchanged in uniform and linear changes of the brightness and contrast of the window:

(18) $$\begin{array}{c} C_{NCC}\left(p,d\right)=\frac{\sum_{i=1}^{n}\sum_{j=1}^{m}\left(\left(I_{L}\left(p\right)-{\bar{I}}_{L}\right)\left(I_{R}\left(p-d\right)-{\bar{I}}_{R}\right)\right)}{\sqrt{\sum_{i=1}^{n}\sum_{j=1}^{m}{\left(I_{L}\left(p\right)-{\bar{I}}_{L}\right)}^{2}\sum_{i=1}^{n}\sum_{j=1}^{m}{\left(I_{R}\left(p-d\right)-{\bar{I}}_{R}\right)}^{2}}}. \end{array}$$

**Figure 3:**
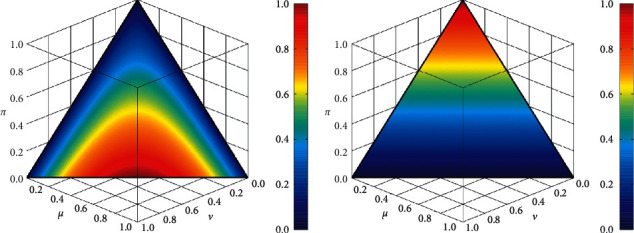
Fuzzy entropy comparison.

Figure 3 shows a schematic representation of the relative entropy differentiation in the applied sample.

The concept of entropy is mainly based on the difficulty of distinguishing between a set and its complement, so the less the set differs from its complement, the vaguer it is. Therefore, there is a specific reason for the percentage of anomaly that characterizes it. In this sense, each probabilistic set is generated by randomizing the degree of participation of each element of its definition field separately. For this purpose, a probabilistic space is introduced and a random variable is assigned to each element with values between the space of the measure of similarity of the factors under consideration [7, 22, 23].

**Figure 4:**
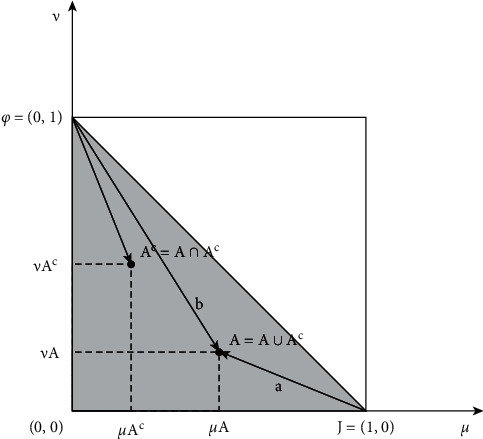
Geometric similarity using fuzzy Euclidean distance.

Figure 4 presents the methodology of accurate geometric determination of the fuzzy anomaly of the samples, utilizing the measurement of their Euclidean vague similarity [24]. The distance between two fuzzy sets is defined to be the regular Euclidean distance between the two corresponding vectors.

**Figure 5:**
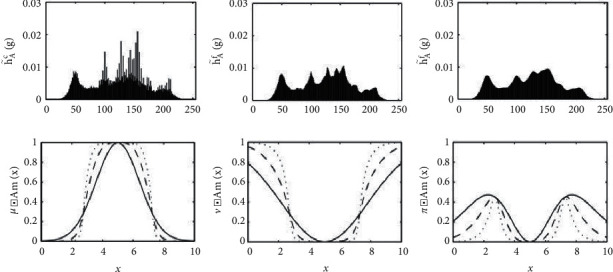
Similarity measure using image features.

The general differences that can identify local or global anomalies were also investigated based on the proposed architecture and the characteristics of the data under consideration, as shown in Figure 5.

Finally, Table 1 presents typical snapshots of the process of using the VFA algorithm and the success rates we achieved.

When the increase or decrease of the anomaly is not abrupt, the extra time points included in the control intervals have a relatively high probability of redefinition with higher accuracy. However, if we have a sharp increase or decrease in probability, the intervals may include times when the activity may show less categorization accuracy. This observation is related to the inherent noise in the dataset, resulting in the fluctuation of the algorithm tendency. In general, however, the finding is that the proposed system can evaluate and categorize with significant accuracy anomalies in a highly complex environment.

The computational complexity is linearly dependent on the sequence length, which means inference is fast and scalable to very large files. All experiments were performed in the Google Colab environment using a GPU processor. To avoid high overhead and achieve timely model convergence, it was necessary to train the proposed system using a relatively small but at the same time satisfactory batch size. Due to the overuse of memory, the heuristic algorithm was used to calculate the digital variation and optimize these values while calculating the fuzzy sample entropy and Euclidean fuzzy similarity. It turns out that these methods are suited to perform the computation of extremely complex processes.

## 5. Conclusions

The detection and timely evaluation of abnormalities in machine vision systems allow the industrial sector to make innovative leaps. This logic is in line with Industry 4.0 and the vision for innovative approaches in modern industry. In this work, we presented a machine vision system that contributes to the efficiency of the new ecosystem of Industry 4.0. It is an intelligent system for identifying anomalies in advanced gearbox assembly systems. Specifically, we presented the VFA methodology whereby using fuzzy entropy and Euclidean fuzzy similarity measurement, we maximized the possibility of using nonlinear transformation through deterministic functions, thus creating an entirely realistic and highly reliable machine vision detection system.

The evaluation of the methods was carried out in a highly complex cybersecurity scenario, where cybercriminals could modify the assembly parameters of the production mechanisms. This fact is not perceived by the other sensors connected to the production system. Utilizing the most advanced machine vision techniques and fuzzy logic methodologies, the proposed method has achieved very high success rates, creating serious expectations for additional cybersecurity applications.

Significant progress could be made in hardening the system with methods of intuitive fuzzy logic. In addition to similarity measures between samples, dissimilarity measures could also be measured, thus making the system even more sensitive and realistic. Also, an extension of the proposed method could study the system's operation in an inversely proportional manner, where two VFAs would operate as opposed to the parallel detection of anomalies.

## Figures and Tables

**Table 1:** Performance evaluation of the proposed algorithm.

	Precision	Recall	*F*-measure	Anomaly score
VFA_Instance_1	88.9	89.0	89.0	0.12
VFA_Instance_2	86.7	87.1	86.8	0.18
VFA_Instance_3	92.1	92.0	92.1	0.08
VFA_Instance_4	89.9	89.9	89.9	0.11
VFA_Instance_5	90.3	90.2	90.2	0.10
VFA_Instance_6	84.5	84.7	84.8	0.22
VFA_Instance_7	88.3	88.3	88.2	0.13
VFA_Instance_8	94.2	94.2	94.0	0.06
VFA_Instance_9	95.1	95.0	95.1	0.05
VFA_Instance_10	93.4	93.4	93.4	0.06
Average score	90.34	90.38	90.35	0.11

## Data Availability

The data used to support the findings of this study are available from the corresponding author upon reasonable request.
